# Scotch Pine Cones-Derived Hard Carbon as an Anode Material for Sodium-Ion Battery Applications

**DOI:** 10.1021/acsomega.4c10363

**Published:** 2025-03-11

**Authors:** Y. Bhaskara Rao, Ola Sundman, Michael Holmboe, Naser Tavajohi, C. André Ohlin

**Affiliations:** Department of Chemistry, Umeå University, Umeå 90187, Sweden

## Abstract

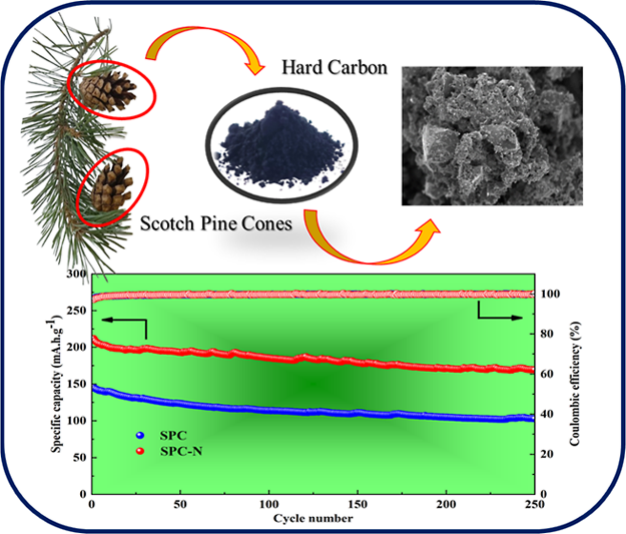

A biobased anode material for sodium-ion batteries (SIBs) was prepared through the simple pyrolysis of Scotch pine cones (*Pinus sylvestris*, SPC), followed by a heteroatom doping modification. The resulting nitrogen-doped hard carbon exhibited a high reversible capacity of 273 mA·h·g^–1^ at a current density of 25 mA·g^–1^ compared to the undoped material (197 mA·h·g^–1^). X-ray diffraction analysis shows that the produced hard carbon from the biomass is highly amorphous in nature, and high-resolution transmission electron microscopy images reveal the presence of localized graphite-like structures that are found to be beneficial for the storage and transport of Na^+^ ions during charging/discharging. Experimental results demonstrated that the increased specific surface area (*S*_BET_ = 424 m^2^·g^–1^), high micropore volume (0.177 cm^3^·g^–1^), and expanded interlayer spacing (>3.7 Å) and a high Na^+^-ion diffusion coefficient (3.08 × 10^–16^ cm^2^·s^–1^) facilitated the diffusion of sodium ions, leading to a high capacity retention of 80% after 250 cycles for the SPC-N material over the undoped one, SPC (71%). This study highlights the potential of low-cost, widely available biobased Scotch pine cones as an alternative anode material to enhance the sustainability of SIB production.

## 1 Introduction

Sodium-ion batteries (SIBs) hold great promise for large-scale energy storage systems because of the high natural abundance of sodium, balanced global distribution, and significantly lower cost in comparison with lithium.^1−3^ However, using metallic sodium as the anode in SIBs can pose safety risks both during production and usage due to the low melting point (97.7 °C) and high reactivity.^4^ Therefore, identifying alternative anode materials that meet the industrial requirements of low cost, high performance, and sustainability is crucial for advancing the commercialization of SIBs.^5^ In recent years, diverse materials have been explored as potential anode materials for SIBs, including alloys, carbonaceous systems, organic compounds, and transition metal oxides.^6,7^ Each class of these materials possesses distinct advantages and disadvantages (Table 1).

**Table 1:** Merits and Demerits of Various Anode Materials for SIBs

anode material	advantages	drawbacks	examples
metal oxides^7^	high volumetric energy density	low initial Coulombic efficiency	Na_2_Ti_3_O_7_, Fe_2_O_3_, and TiO_2_
		large potential hysteresis	
		inferior cyclability	
organic compounds^7^	structural flexibility	high solubility	Na_2_C_8_H_4_O_4_ and Na_2_C_6_H_2_O_4_
	possible multielectron reaction	sluggish kinetics	
alloys^7^	high theoretical capacity	severe volume expansion	SnSb and Sn_4_P_3_
	low redox potentials		
carbonaceous materials^13^	negligible volume change	poor cycling stability	Hard Carbon
	structural stability		
	low cost		

Among them, carbonaceous anode materials offer superior cycle performance due to their limited volume change and structural stability, which set them apart from alloy and conversion anodes. Conventional graphite has an interlayer spacing of *ca* 0.335 nm and, due to this reason, Na^+^ ions cannot be intercalated into the layers because of their larger ionic radii.^8^ However, hard carbons (HCs) have large interlayer spacing (>0.37 nm) which can accommodate the Na^+^-ion insertion in a range of chemically and physically dissimilar storage sites. Further, HCs have more disordered structures, a higher concentration of defects, a higher content of heteroatoms, a larger distance between the graphitic layers, and more closed pore structures than soft carbons.^9,10^ Moreover, the specific capacity of HC in SIBs can approach that of commercial graphite anodes of lithium-ion batteries (LIBs), even though HC has a lower specific capacity than that of chalcogen-based materials and alloys.^11,12^ Generally, HCs can be derived from different precursors, such as carbohydrates, such as sucrose, glucose, resins, polymers, and different biowastes, such as orange peel, banana peel, and lotus stems. Among them, the use of biomass to produce HC is economically and environmentally beneficial, as it makes many waste products, such as lignin, banana peels, and apricot shells, recyclable.

Because of the variations in their chemical compositions, HC obtained from different biomass precursors often exhibits distinct electrochemical properties. A recent analysis reviews the different synthesis conditions and preparation techniques governing the physical characteristics of HC material made from various biomasses.^13^ For instance, Demir et al. demonstrated the use of apricot shells for the synthesis of HC treated at 1000 °C, which delivered a high discharge capacity of 184 mA·h·g^–1^ at 0.1 C over 250 cycles.^14^ A biocarbon anode from lychee seeds, prepared at 500 °C, exhibited an exceptional electrochemical performance against sodium metal, as evidenced by its specific capacity of approximately 146 mA·h·g^–1^ at 0.2 A g^–1^ current density for 100 cycles.^15^ On the other hand, the best rate performance of 172 mA·h·g^–1^ at 200 mA·g^–1^ and outstanding cycling performance with 96% capacity retention after 50 cycles is displayed by HC prepared at 1300 °C from sorghum stalk.^16^ Avocado peel HC heat-treated at 1100 °C exhibited an excellent reversible capacity of 352.55 mA·h·g^–1^ at 0.05 A·g^–1^, a high capacity retention of >90%, and a Coulombic efficiency of 99.9% even after 500 cycles.^17^

HC materials are thus very attractive for use in SIB anodes as they are widely available, low-cost, and environmentally friendly and they offer much better sodium-ion storage than graphite. Challenges remaining include the improvement of cycle life, however, which is still far less than that required to achieve grid-scale energy storage.^13^ In this study, HC is derived from the cones of Scotch pine (*Pinus sylvestris*), a new biomass, which is the most extensively dispersed conifer in the world, growing from latitude 70° to 37° N and from 8° W in Spain to 141° E in Russia. All of the European Union (EU) member states have natural forests or plantations of this species, which is very important for producing timber, especially in the Nordic region.^18^

Despite the advantages in terms of availability, low cost, and sustainability, the performance of HC in SIBs remains suboptimal, necessitating further modifications. One promising approach to enhance the HC performance is defect engineering, particularly through heteroatom doping (e.g., with nitrogen, sulfur, boron, and phosphorus).^13,19^ N doping could potentially improve the ability of the HC to store Na^+^ ions and increase ionic conductivity by decreasing energy barriers and facilitating the transport of Na^+^ ions within the HC matrix.

In the present study, melamine, as a nitrogen source, is combined with the HC produced from Scotch pine cones to improve the electrochemical performance of SIBs compared to the untreated HC. In general, the processing temperatures of biomass to produce HC are in the range of 600–1500 °C, and it was earlier reported that as the carbonization temperature increases, graphitic domains increase and the *d* spacing between graphene layers decreases. On the other side, increase in carbonization temperature leads to an increase in open pore size but a decrease in total open pore volume. Closed pore volume also decreases at higher carbonization temperatures.^13^ In view of all the above, an optimal pyrolysis temperature (800 °C) and melamine to HC *weight* ratio of 1:1 are considered in the present study to evaluate the performance of Scotch pine cone-derived HC electrode material.^20,21^ The present study reveals the promising electrochemical performance of the HC anode material in Na-ion batteries, supporting the previous hypothesis.

Further, the amorphous nature of the HC materials is confirmed by using the X-ray diffraction (XRD) technique. The presence of D and G bands is analyzed through Raman spectroscopy. The morphology and microstructure of the HC anode materials are investigated by scanning electron microscopy (SEM) and high-resolution transmission electron microscopy (HRTEM). Finally, the electrochemical performance of both materials is analyzed through galvanostatic charge–discharge studies, cyclic voltammetry (CV), electrochemical impedance spectroscopy (EIS), and quantitative repartition of capacitive and diffusive behaviors.

## 2 Experimental Section

### 2.1 Material Synthesis

Scotch pine (*P. sylvestris*) cones were collected and soaked in dilute HCl (1.2 M) for 12 h, filtered, and then dried in an oven at 100 °C for 4 h to remove any impurities. The dried product was smashed into small pieces and then directly pyrolyzed at 800 °C for 8 h under a N_2_ atmosphere. The obtained product was ball-milled (400 rpm; 12, 8, and 5 mm balls) for 4 h to get a fine and smooth black powder. The powder was then again washed with diluted HCl (1.2 M) to dissolve any metal particles, filtered, and further dried at 100 °C in an oven. The HC derived from the Scotch pine (SPC) was further mixed with the melamine (N source, 1:1 by weight). The weight ratio of 1:1 was chosen as this has been shown to be the optimal ratio.^21,22^ It was then heat-treated at 800 °C for 2 h under a N_2_ atmosphere to obtain the final N-doped HC powder, referred to as SPC-N.

### 2.2 Material Characterization

XRD was carried out on a PANalytical Xpert^3^ powder X-ray diffractometer with Cu-*K*α radiation. The samples were scanned in the 2θ range of 10–80° with a step size of 0.02626°. Raman spectra were recorded using a Renishaw Qontor instrument (Renishaw Plc, UK), running WiRe (version 5.3). A 532 nm solid-state laser with a maximum nominal power of 50 mW was used through a 20× lens in normal confocality mode. One percent laser power was used with 1s exposure time in static mode, centered at 1300 cm^–1^. SEM images were collected on a Zeiss Merlin Schottky FEG-SEM instrument with a GEMINI II column. The acceleration voltage (or electron high tension, EHT), the prober current, and working distance are noted on the respective image. HRTEM images were taken using a Talos L120C (FEI, Eindhoven, The Netherlands) operating at 120 kV. Micrographs were acquired using a Ceta 16 M CCD camera (FEI, Eindhoven, The Netherlands), equipped with TEM Image and Analysis software ver. 4.17 (FEI, Eindhoven, The Netherlands). The X-ray Photoelectron Spectroscopy (XPS) spectra were obtained using a Kratos Axis Ultra DLD electron spectrometer with a monochromated Al-*K*α source operating at 150 W. Wide spectra were acquired with an analyzer pass energy of 160 eV, while individual photoelectron lines were measured at 20 eV. Powder samples were gently hand-pressed into pellets directly on the sample holder using a clean nickel spatula. Since the samples were conductive, the spectrometer charge neutralization system was not used. Spectral processing was performed by using Kratos software. N_2_ physisorption measurements were conducted on a Micromeritics TriStar 3000 porosimeter. Adsorption–desorption isotherms were recorded at −196 °C after the samples had been outgassed at 120 °C for 3 h. The specific surface areas were collected by the Brunauer–Emmett–Teller (BET) method, and the pore volumes were calculated from desorption isotherms. The pore size distributions were estimated using the Barret, Joyner, and Halenda (BJH) algorithm using ASAP-2010 software.

### 2.3 Electrochemical Characterization

The electrochemical tests were carried out using CR-2032 coin-type half-cells assembled in a nitrogen-filled glovebox (MBraun-MB10—compact) with O_2_ and H_2_O levels <0.5 ppm. All the electrodes were prepared by mixing the active material, Super P (Thermo Scientific), and sodium carboxy methyl cellulose (CMC—MedChemExpress) binder in a 80:15:5 weight ratio in deionized water. The mixed slurry was then applied onto the surface of a copper foil (TMAXCN; 14 mm diameter, 0.1 mm thick) current collector and then dried at 50 °C. The average mass loading of the active material was approximately 2–3 mg·cm^–2^. A glass microfiber filter (Whatman, grade GF/F; 19 mm diameter) was used as the separator, and 1 M NaClO_4_ (Thermo Scientific) in ethylene carbonate (EC, from AmBeed) and dimethyl carbonate (DMC, from TCI; 1:1 v/v) was used as the electrolyte. The electrochemical measurements were carried out using sodium metal as the counter/reference electrode. CV curves at different scan rates and EIS in the frequency range of 0.1 Hz–1 MHz were recorded using a Gamry 1010E interface workstation. The galvanostatic charge–discharge measurements were performed by using a NEWARE CT-4008 battery tester in the voltage window of 0.01–2.5 V (vs Na^+^/Na) under different current densities. All the electrochemical measurements were performed at room temperature (20–23 °C).

## 3 Results and Discussion

**Figure 1:**
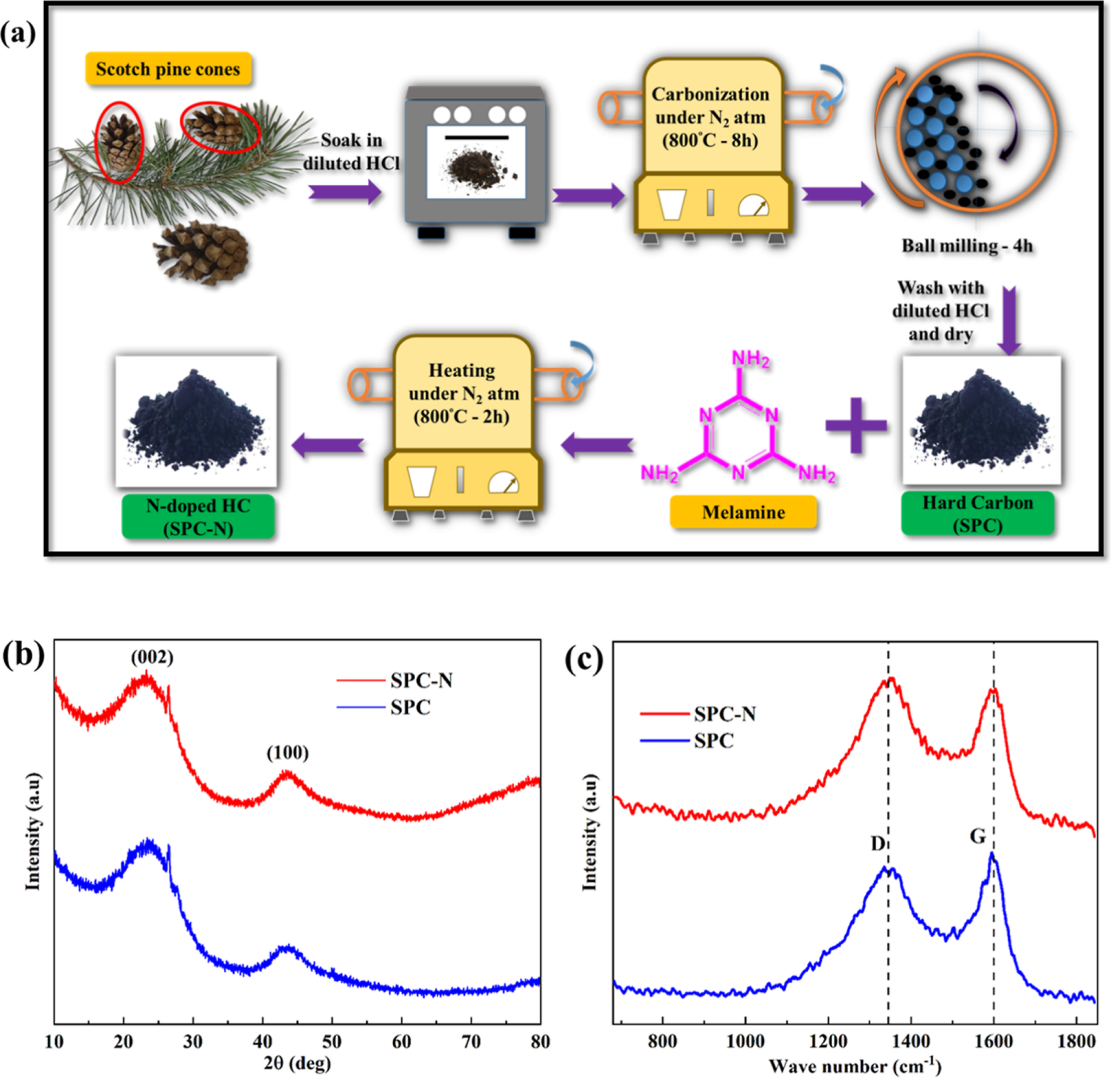
(a) Preparation process of HC from Scotch pine cones, (b) XRD patterns, and (c) Raman spectra of SPC and SPC-N materials.

The schematic pathway of the preparation of HC starting from the biomass, Scotch pine cones, and mixing of melamine (N doping) with the HC is summarized in Figure 1a, while Figure 1b shows the XRD patterns of SPC and SPC-N samples. Here, the HC is heat-treated at 800 °C and mixed with the optimal melamine content (1:1 weight) according to the previous studies.^20,21,23^ Each sample displays two broad diffraction peaks at 2θ of ∼23° and ∼44° corresponding to the crystallographic planes of (002) and (100), respectively, which indicates that the synthesized carbon materials have amorphous structures.^24^ The *d* spacings (*d*_002_) of the two samples are calculated using Bragg’s equation and are found to be 0.379 and 0.384 nm for SPC and SPC-N, respectively. The *d* spacing increases slightly on N doping and these values are still larger than that of graphite (0.335 nm) which is beneficial for the storage of Na^+^ ions.^25^

The effect of N doping on the degree of graphitization is studied with the help of Raman spectra (Figure 1c). The two characteristic peaks appear at ∼1340 cm^–1^ and ∼1600 cm^–1^, respectively, corresponding to the D peak which represents the lattice defects and the G peak, representing the stretching vibration mode of the graphite lattice, separately.^26^ The intensity ratio (*I*_D_/*I*_G_) of the D to G peaks can be helpful for determining the degree of amorphous structure in HC samples, and the values are found to be 0.92 and 1.06 for the SPC and SPC-N materials, respectively. It is clear that the value of I_D_/I_G_ is close to unity for both materials, which indicates that the ordered graphene sheets are highly defective.^27^ Here, the SPC-N sample shows a somewhat higher *I*_D_/*I*_G_ ratio, which indicates that it has a more amorphous nature, which introduces more structural defects into the HC sample and thus affects the electrochemical properties of the SPC electrode.

**Figure 2:**
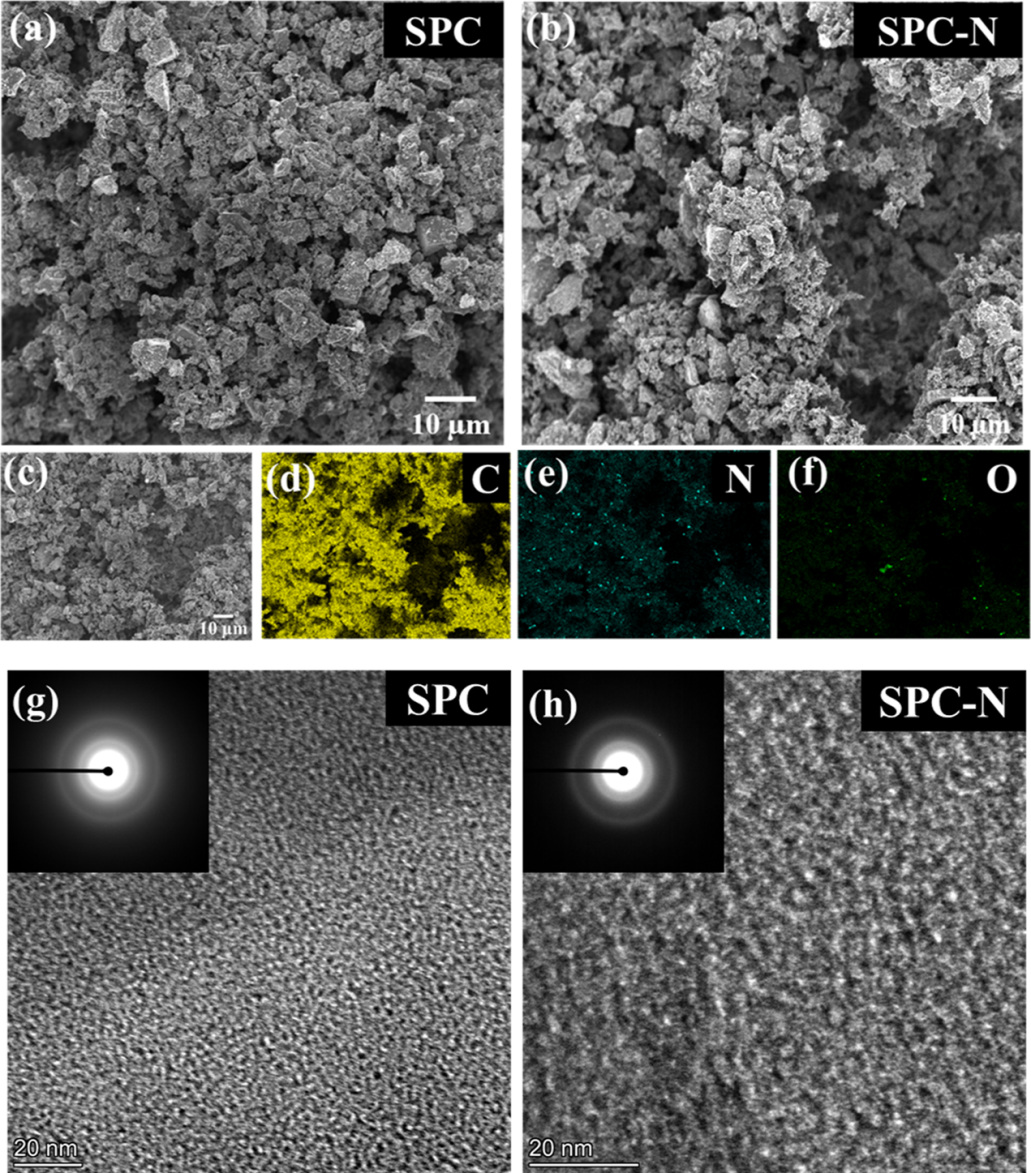
SEM images of SPC (a) and SPC-N (b) materials, (c–f) EDX elemental mapping images of the SPC-N material, and HRTEM images of SPC (g) and SPC-N (h) materials.

The SEM images of SPC and SPC-N are shown in Figure 2. Both HC materials show similar morphology in the SEM images, irrespective of doping. The carbonized Scotch pine cones yield irregularly shaped micrometer-sized carbon particles (Figure 2a,b), and the porous structure inherited from the pine cones leads to surface area enhancement, which further facilitates the penetration of electrolytes. This type of diffusion can enhance the electrochemical and Na^+^-ion storage capabilities of the material by reducing the diffusion distance of the sodium ions. The electron-dispersive X-ray spectra (EDX) elemental mapping images (Figure 2c–f) show the presence of carbon, nitrogen, and oxygen well-dispersed throughout the SPC-N sample. Further, local ordered turbostratic domains are observed in the HRTEM images of both samples, which are shown in Figure 2g,h.

**Figure 3:**
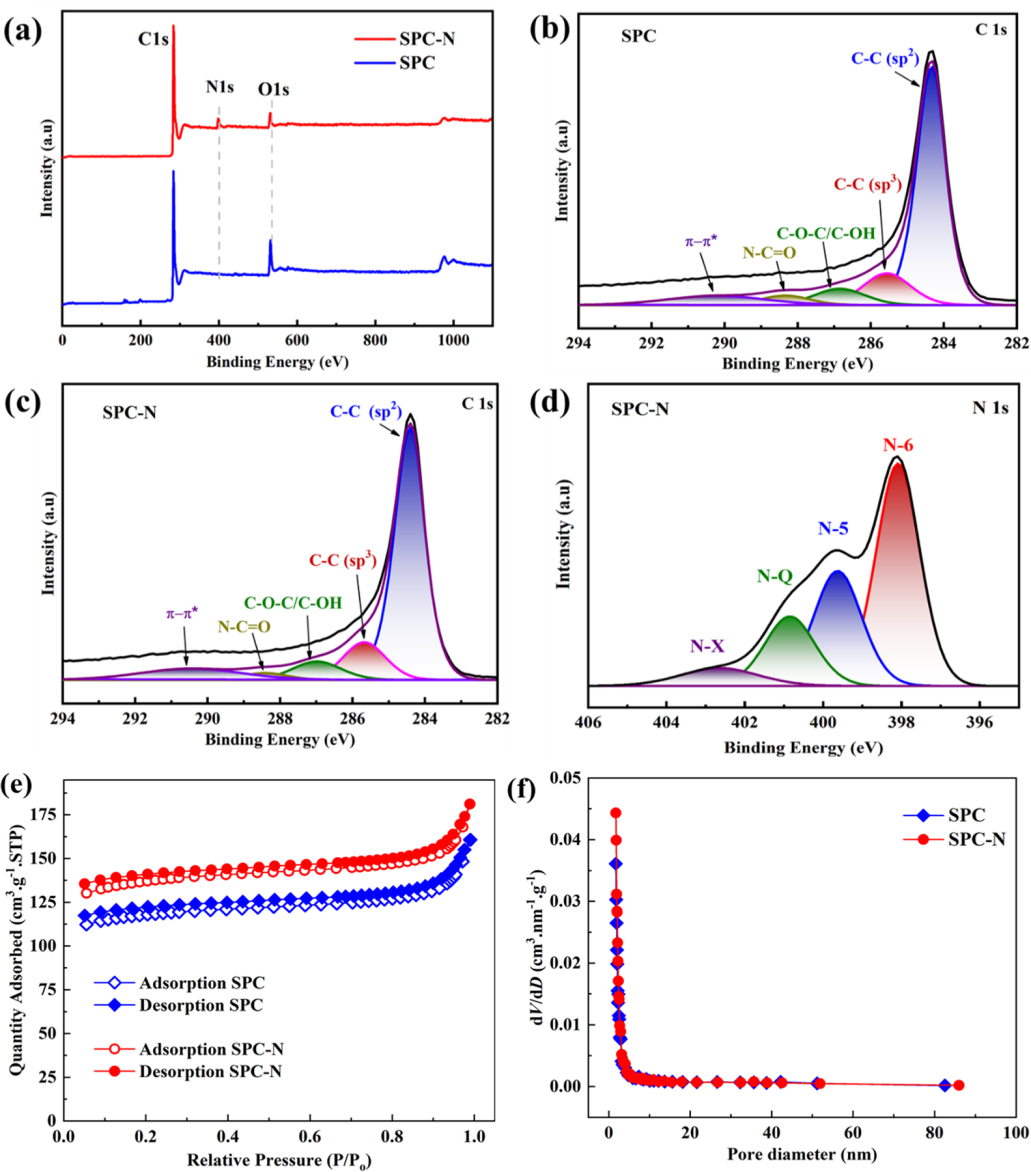
(a) XPS survey spectra of SPC and SPC-N samples, C 1s spectra of (b) SPC and (c) SPC-N, (d) N 1s spectra of the SPC-N sample, (e) N_2_ adsorption/desorption isothermal curves, and (f) pore size distribution function of SPC and SPC-N materials.

XPS was performed to investigate the various chemical bonding configurations in both the SPC and the SPC-N samples, the results of which are shown in Figure 3. The survey spectra of both samples found strong peaks of C 1s and O 1s at binding energies of around ∼284 eV and ∼531 eV, respectively. A signal at ∼400 eV is attributed to N 1s in the SPC-N material; however, no such signal is found in the undoped material, SPC. The C 1s spectra for both samples exhibit five peaks with binding energies close to 284, 285, 287, 288, and 290 eV, corresponding to the chemical bond configurations of C–C (sp^2^), C–C (sp^3^), C–O–C/C–OH, N–C=O, and π–π*, respectively (Figure 3b,c).^19^ In general, nitrogen adopts three main bonding configurations within the carbon matrix: pyrrolic (N5)—situated in the five-membered rings of carbon, pyridinic (N6)—situated in the six-membered rings of carbon, and graphitic or quaternary (NQ)—situated in the six-membered rings without any surrounding vacancies.^13^ Here, the signals of N6, N5, and NQ content in the sample SPC-N are observed at energies of *ca* 398 eV, 399 eV, and 401 eV, respectively, which is shown in Figure 3d. In addition, a weak and broad signal at *ca* 403 eV corresponds to the N-X (pyridine-N-oxide) configuration.^28^ It was earlier reported that N6 and N5 can induce defects in the carbon structure, leading to improved electrochemical conductivity and sodium storage, while NQ facilitates electron transport, which contributes to the improved rate performance.^29,30^

N_2_ adsorption–desorption isothermal curves from BET analysis reveal the properties of porous materials in terms of porosity, specific surface area, and pore size distribution. Isothermal curves of the SPC and SPC-N samples are shown in Figure 3e. A higher BET specific surface area of 424 m^2^·g^–1^ is found for SPC-N, compared to that of undoped SPC (373 m^2^·g^–1^), suggesting an enhancement in the electrochemical behavior of the N-doped sample. Furthermore, the pore size distributions are estimated by the Barrett–Joyner–Halenda method (Figure 3f) and an average pore size of *ca* 2.5 nm is found for both samples. It was earlier reported that the diffusion of nitrogen functional groups into the deeper pores of carbon can affect the microporosity of the material.^31^ Thus, the micropore volume of the undoped material SPC is recorded as 0.155 cm^3^·g^–1^, while for the SPC-N material, it is found to be 0.177 cm^3^·g^–1^.

**Figure 4:**
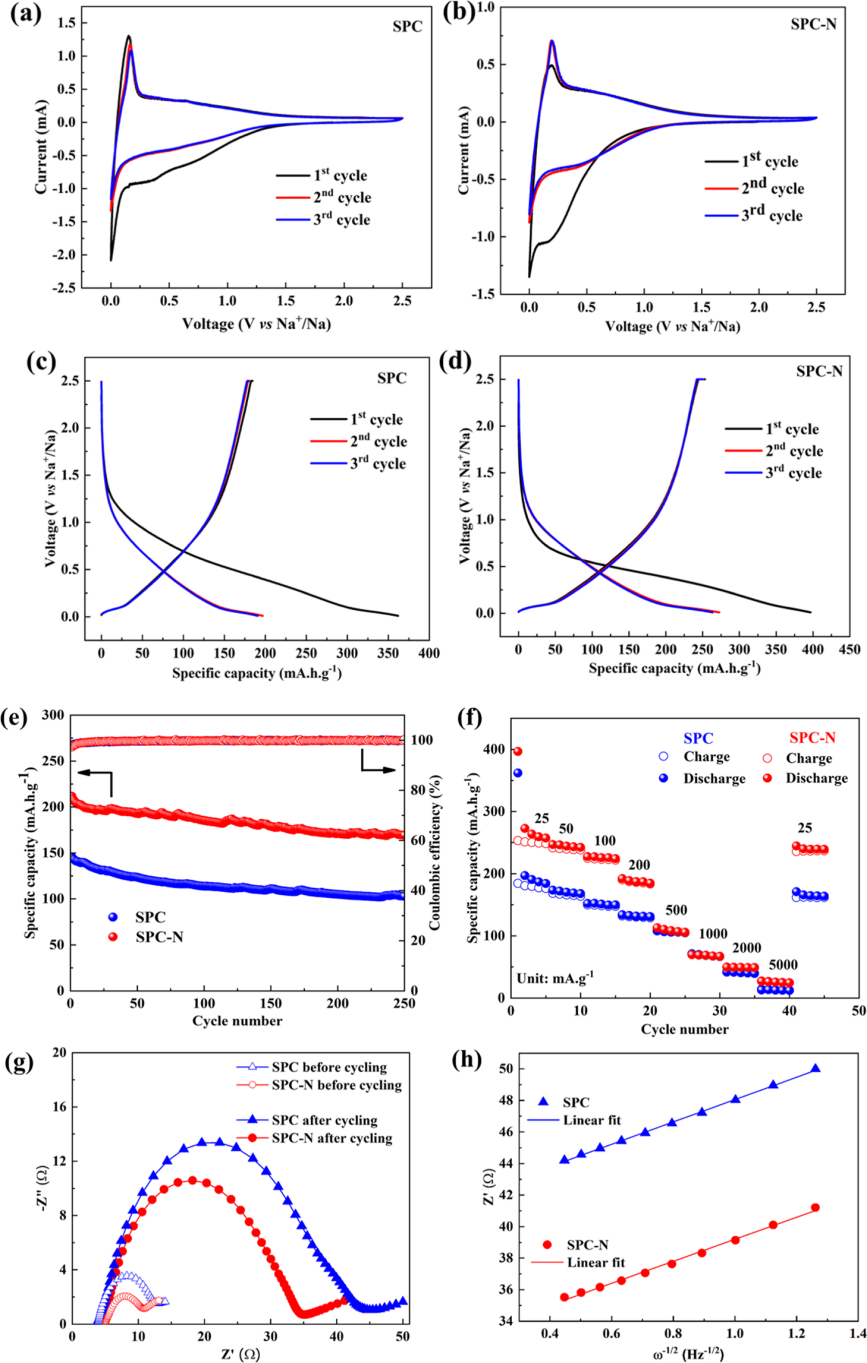
Cyclic voltammograms of SPC (a) and SPC-N (b) materials at a scan rate of 0.2 mV·s^–1^ and charge–discharge profiles of SPC (c) and SPC-N (d) materials at a current density of 25 mA·g^–1^, (e) cycling stability at a current density of 200 mA·g^–1^, (f) rate performance at various current densities, (g) complex impedance curves of SPC and SPC-N electrodes before and after 10 cycles, and (h) plot of real part of impedance, Z′ with ω^–1/2^ at lower frequencies of SPC and SPC-N materials.

CV studies were carried out at a scan rate of 0.2 mV s^–1^ in the potential window of 0.0–2.5 V *vs* Na^+^/Na in order to understand the electrochemical performance of both samples as shown in Figure 4a,b. During the initial cathodic scan, both samples exhibited an irreversible reduction peak at ∼0.25 V, which disappeared during subsequent cycles. This was caused by the irreversible reaction of the electrolyte and the formation of a solid–electrolyte interface (SEI) layer on the electrode surface.^32^ However, excellent stability is maintained during subsequent cycles, which is evident from the well-overlapped CV curves. Also, a pair of redox peaks are present at *ca* 0.1 V, which are attributed to the insertion and extraction process of sodium in both samples. The initial three discharge/charge profiles of SPC and SPC-N samples at a current density of 25 mA g^–1^ over a voltage range of 0.01–2.5 V are shown in Figure 4c,d. The charge/discharge curves of the materials, SPC and SPC-N, consist of two distinct regions: (i) sloping (above 0.1 V) and (ii) plateau (below 0.1 V) regions.^33^^,^^34^ It is known since earlier that the sloping region results from the intercalation of the Na^+^ ions into the parallel graphitic layers of the HC, while the plateau region is obtained due to the filling of the Na^+^ ions into the nanopores of the HC.^35^^,^^36^ The two regions have different capacities for both of the electrode materials (Figure 4c,d). In addition, the SPC-N material often exhibits the highest plateau and sloping capacities compared to that of the undoped material due to its large pore volume (see BET Figure 3e,f) and expanded interlayer spacing (*d*_002_ = 0.384 nm) after doping with nitrogen. A higher irreversible discharge capacity of 396 mA·h·g^–1^ is observed for the SPC-N sample compared to that of the undoped sample, SPC (362 mA·h·g^–1^). Moreover, the deintercalation of sodium ions results in charge capacities of 184 and 253 mA·h·g^–1^ for SPC and SPC-N electrodes, respectively. The high, irreversible capacity loss in the first cycle is attributed to the electrode–electrolyte interaction phenomenon which results from the formation of a stable SEI layer on the surface of the anode material in line with the CV results (Figure 4a,b).^37^ Thus, the SPC and SPC-N samples exhibited low initial Coulombic efficiencies of 51 and 64%, respectively. However, the materials both delivered high, reversible discharge capacities of 273 mA·h·g^–1^ (SPC-N) and 197 mA·h·g^–1^ (SPC), respectively, and the SPC-N material delivered a higher reversible discharge capacity compared to that of the undoped material, SPC, due to the availability of more active sites for Na^+^-ion transport and storage after doping nitrogen into the HC matrix. Thus, the cyclic performance is improved by recording a high Coulombic efficiency of >92% from the second cycle.

The long-term cycling stability of SPC and SPC-N electrodes at a current density of 200 mA·g^–1^ is further evaluated (Figure 4e). The undoped sample, SPC, showed an initial specific discharge capacity of 145 mA·h·g^–1^, and it delivered a discharge capacity of 103 mA·h·g^–1^ after the 250th cycle. However, the SPC-N material exhibited a higher initial discharge capacity of 211 mA·h·g^–1^ and delivered a discharge capacity of 169 mA·h·g^–1^ after 250 cycles. Here, the N-doped sample exhibited a good capacity retention of 80%, compared to the undoped sample (71%) indicating the high durability of the electrodes for SIBs. Thus, the SPC-N results demonstrate the importance of the presence of nitrogen in enhancing the energy storage due to the expanded interlayer spacing (*d*_002_) owing to the doping of heteroatoms like nitrogen into the HC matrix.^29^Figure 4f shows the rate capability of SPC and SPC-N materials at various current densities from 25 to 5000 mA·g^–1^. The SPC-N material delivered the highest discharge capacities of 273, 247, 227, and 192 mA·h·g^–1^ at current densities of 25, 50, 100, and 200 mA·g^–1^, respectively. On the other hand, the SPC electrode exhibited comparatively lower discharge capacities of 197, 173, 152, and 134 mA·h·g^–1^ at the same current densities. However, at higher current densities, a similar performance is exhibited by both the materials, owing perhaps to preference of Na^+^ ions to diffuse toward the defect sites rather than the graphene layers at fast current rates.^38^ The cycling at higher current rates does not damage the structure of both SPC electrodes permanently, since a significant amount of specific capacity was still retained even when the current density is reverted to 25 mA g^–1^, which represents a better rate performance for both samples. Furthermore, Figure 4g shows the EIS of the SPC and SPC-N electrodes, which is used to elucidate the electrochemical kinetics. The compressed semicircle at the high-medium frequency region is ascribed to the charge transfer resistance (*R*_ct_), and an inclined spike at lower frequencies corresponds to the Warburg impedance (*Z*_w_).^39^ After fitting with the equivalent circuit, the *R*_ct_ values of SPC and SPC-N samples are found to be 8.4 and 5.7 Ω, respectively, before cycling, whereas the high charge transfer resistances of 37.7 and 29.5 Ω are observed after ten cycles for the same samples. The increased *R*_ct_ values during the cycling may be due to the formation of an interfacial layer between the electrode and the electrolyte.^40^ The SPC-N sample records a lower charge transfer resistance compared to that of the undoped one, which indicates an improved Na^+^-ion conduction in the N-doped material. This is well-understood by calculating the Na^+^-ion diffusion coefficient with the help of the formula in eq 1,^38^
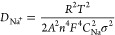
1Here, *A* is the surface area of the electrode, *n* is the number of electrons transferred in the electrochemical redox reaction, *F* is the Faraday constant, *C*_Na_ is the molar concentration of Na^+^ ions, *T* is the absolute temperature, *R* is the universal gas constant, and σ is the Warburg factor. By using the relationship in eq 2,^41^
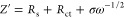
2the slope (σ) can be calculated by plotting a graph between *Z′* and ω^–1/2^ (Figure 4h). As a result, the SPC-N electrode possesses a high sodium ion diffusion coefficient of 3.08 × 10^–16^ cm^2^·s^–1^ compared to that of the SPC electrode (2.98 × 10^–16^ cm^2^·s^–1^). This indicates that N doping into the HC matrix can effectively accelerate the Na^+^ diffusion kinetics and thus improve the electrochemical performance of the HC material. The electrochemical performances of various HC electrodes derived from plant-based biomass are shown in Table 2. Here, the present material, SPC-N, delivers a high capacity retention (80%) at higher current density (200 mA·g^–1^) which is prepared at a comparatively low processing/pyrolysis temperature (800 °C) compared to some other biomass-derived HCs reported in the literature.

**Table 2:** Electrochemical Performance of Various Biomass-Derived HC Electrodes for SIB Applications

carbon source	pyrolysis temperature (°C)	current density (mA·g^–1^)	specific capacity (mA·h·g^–1^)	capacity retention (%)	current density (mA·g^–1^), number of cycles	ref
apricot shell	1000	0.1C	170	43	0.1C, 250	(14)
lychee seeds	500	50	400	65	200, 100	(15)
sorghum stalk	1300	20	241	96	20, 50	(16)
avocado peels	1100	50	353	90	3500, 500	(17)
corn cob	1300	1C	275	97	0.2C, 100	(42)
soap nut seeds	800	25	224	60	100, 100	(21)
argan shell	1200	25	333	94	25, 70	(43)
oatmeal	1200	20	272	93	20, 100	(44)
Scotch pine cones	800	25	197	71	200, 250	this work
Scotch pine cones (N-doped)	800	25	273	80	200, 250	this work

**Figure 5:**
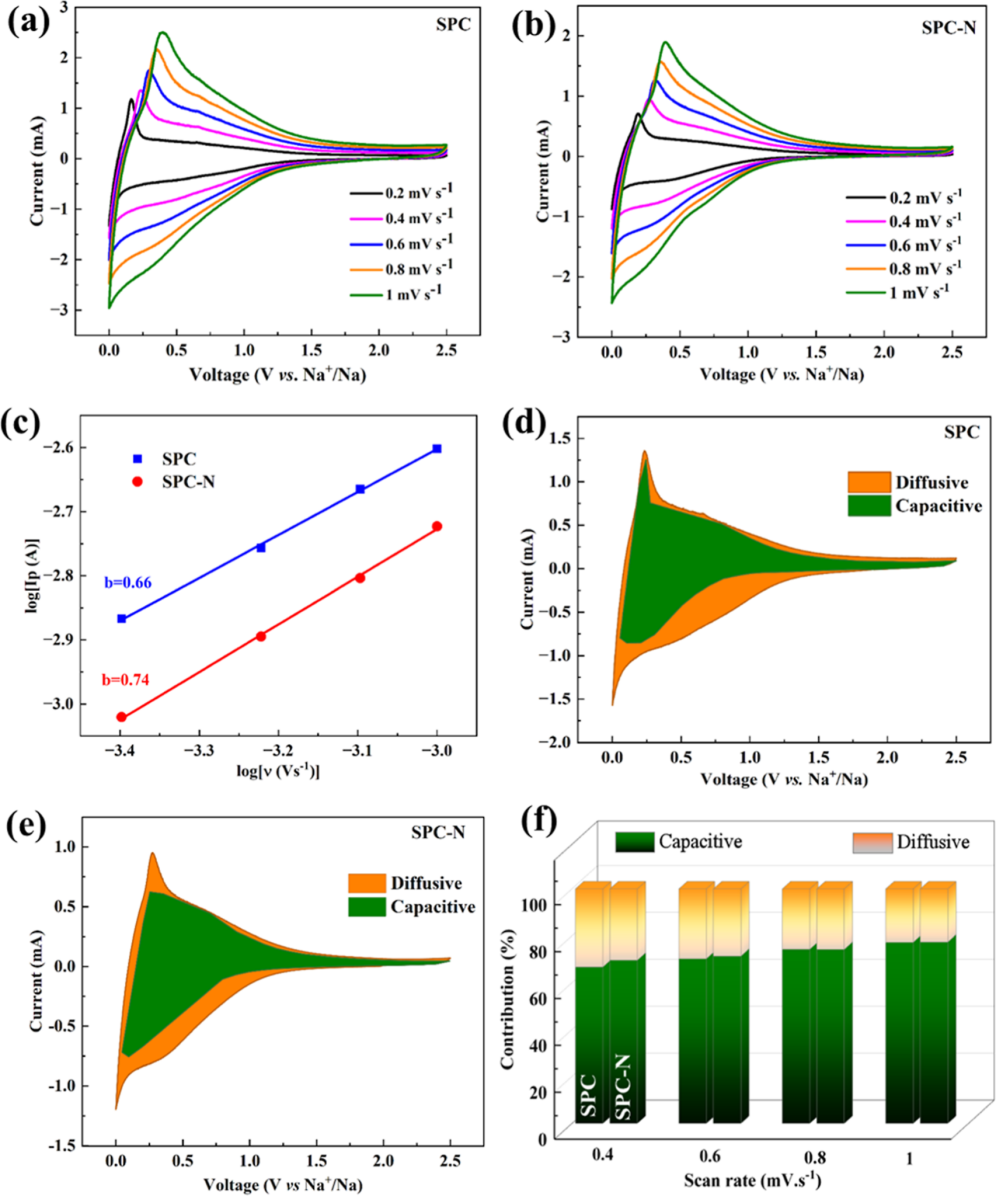
Cyclic voltammograms of SPC (a) and SPC-N (b) samples at various scan rates, (c) plot of the logarithm of peak current (*i*_p_) with the logarithm of scan rate (ν), CV curves showing the capacitive and diffusive contributions for (d) SPC and (e) SPC-N materials, and (f) percentage contributions of the capacitive and diffusive processes at various scan rates for SPC and SPC-N electrodes.

The CV plots of SPC and SPC-N materials at various scan rates are shown in Figure 5a,b. Here, the peak current increases with increasing scan rate in both cases, and the oxidation peak shifts toward a higher potential, indicating smaller polarization of both the SPC and SPC-N anodes.^45^

The electrode kinetics were further investigated by using the power law relationship shown in eq 3
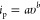
3where *i*_p_ is the peak current, υ is the scan rate, and *a* and *b* are constants obtained from fitting.^21,30^Eq 3 can be rewritten as eq 4
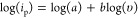
4Here, the slope *b* can be calculated from the straight linear fit of the plot of log(υ) vs log(*i*_p_) (Figure 5c). The capacitive and diffusion-controlled contributions can be characterized using the value of *b*. The storage mechanism is purely capacitive (surface-controlled) if *b* is equal to 1, whereas it is diffusion-limited (charge intercalation) if *b* approaches 0.5. However, both mechanisms contribute to the storage process if *b* lies between 0.5 and 1.^21^ From Figure 5c, the *b* values of SPC and SPC-N electrodes are found to be 0.66 and 0.74, which indicate that the sodium-ion storage mechanism is controlled by both diffusion and capacitive processes.

Additionally, the quantitative repartition of diffusive and capacitive contributions to the overall storage mechanism of SPC and SPC-N samples was obtained according to eq 5
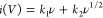
5where *k*_1_ν and *k*_2_ν^1/2^ denote the surface capacitive and the diffusion-controlled contributions, respectively.^46^Figure 5d,e illustrate the capacitive and diffusive contributions on the CV plot of SPC and SPC-N electrodes at a scan rate of 0.4 mV·s^–1^, and it is clear that the percentage of capacitive contribution increases gradually with the scan rate from 0.4 to 1 mV·s^–1^ (Figure 5f). The capacitive surface contribution is a little higher in the case of SPC-N HC due to its highly porous nature (*S*_BET_) resulting from nitrogen doping. This outcome concludes that the HC obtained from the Scotch pine cones has surface functional groups, defect sites, and micropore zones, where Na^+^ ions can readily be adsorbed.

## 4 Conclusions

The HC obtained from Scotch pine cones, a new biomass, demonstrated exceptional electrochemical performance. The N-doped material, SPC-N, in particular, demonstrated encouraging outcomes because of its high specific surface area and wide interlayer spacing. It delivered a higher reversible capacity of 273 mA·h·g^–1^ at a current density of 25 mA·g^–1^ over the undoped one, SPC (197 mA·h·g^–1^). Further, the introduced defects and disorder in the HC matrix led to a significant capacity enhancement along with a high rate performance of 192 mA·h·g^–1^ at 200 mA·g^–1^ compared with that of the undoped material. The N-doped sample also records a remarkable capacity retention of 80% even after 250 cycles, which was attributed to its high Na^+^-ion diffusion coefficient (3.08 × 10^–16^ cm^2^·s^–1^). Thus, the electrochemical performance of the material, SPC-N, is comparable and superior to that of the earlier findings. As an effective anode material in SIBs, we expect that the HC material made from Scotch pine cones offers a wide variety of applications (such as supercapacitors). Thus, the current study contributes to the commercialization of next-generation Na-ion batteries by paving the path for the easy production of HC from a widely accessible, inexpensive bioresource that is both industrially and commercially favorable.
